# Loneliness and Subjective Cognitive Concerns in Daily Life

**DOI:** 10.1093/geroni/igaf122.495

**Published:** 2025-12-31

**Authors:** Martina Luchetti, Damaris Aschwanden, Antonio Terracciano, Angelina R Sutin

**Affiliations:** Florida State University College of Medicine, Tallahassee, Florida, United States; Eastern Switzerland University of Applied Sciences, Institute for Ageing Research, St. Gallen, Sankt Gallen, Switzerland; Florida State University, Tallahassee, Florida, United States; Florida State University College of Medicine, Tallahassee, Florida, United States

## Abstract

Loneliness is associated with increased risk of dementia and poor cognitive functioning in middle-aged and older adults. Limited work has examined how loneliness is associated with subjective cognitive concerns, particularly in the context of everyday life. This study used data from the National Study of Daily Experiences, part of the Midlife in the United States Study to examine the daily association between feeling lonely and self-perceptions of cognitive function. Respondents (n = 1,828; mean age = 56.56; 55.7% females) participated in an 8-day daily assessment reporting on loneliness, cognitive concerns (e.g., memory lapses), and other aspects of daily life. Multilevel linear and binary logistic regressions indicated a significant between- and within-person association between loneliness and subjective cognition. At the between-person level, participants who felt lonelier tended to report more cognitive problems. At the within-person level, on days participants felt lonely (independent of the frequency of those feelings), they also reported more trouble concentrating and were more likely to experience memory lapses. Feeling lonely was also associated with more irritation and interference associated with memory lapses. The observed associations were in general not moderated by socio-demographic factors, remained significant controlling for individual- and day-level social and contextual covariates, and when excluding individuals with depression or anxiety. Results suggest that even transitory feelings of loneliness are associated with poor perceptions of everyday cognitive function, a marker with implications for future risk of cognitive decline.

